# Supplementary material for: Do the effects last? A comparison between internal and external focus of attention instructions on golf putting accuracy over multiple days
Source: PeerJ. 2024 Jul 11;12:e17718. doi: 10.7717/peerj.17718 (PMC11246618; doi:10.7717/peerj.17718)
Supplement: Supplemental Information 1 [file peerj-12-17718-s001.docx]

**Do the effects last? a comparison between internal and external focus of attention instructions on golf putting accuracy over multiple days**

**Supplementary Analyses**

**Preferred Attentional Focus**

Table 1 presents the preferred attentional focus of participants in each of the days of practice. The only significant difference was between Day 3 and Day 4, χ^2^ (4) = .02.

**Table 1**

*Number of participants who preferred each of the attentional focus instructions in each of the days of practice*

|  | Day 1 | Day 2 | Day 3 | Day 4 |
| --- | --- | --- | --- | --- |
| Control | 9 | 8 | 10 | 11 |
| External Focus | 8 | 10 | 14 | 9 |
| Internal Focus | 13 | 12 | 6 | 10 |

**Perception of Attentional Focus that Led to the Best Performance**

Table 2 presents the attentional focus under which participants perceived their best performance. The were no significant differences between days (all χ^2^ values > .05).

**Table 2**

*Number of participants who perceived their best performance in each of the attentional focus instructions*

|  | Day 1 | Day 2 | Day 3 | Day 4 |
| --- | --- | --- | --- | --- |
| Control | 14 | 9 | 10 | 11 |
| External Focus | 10 | 8 | 11 | 8 |
| Internal Focus | 6 | 13 | 9 | 11 |

**Differences between Preferred Focus and Perceived Best Focus**

***Day 1***

The preferred attentional focus and the perceived best attentional focus matched in 20 of the 30 participants (Table 3).

**Table 3**

*Preferred vs. perceived best attentional focus on Day 1. Values represent the number of participants*

|  | Perceived Best Attentional Focus | | |  |
| --- | --- | --- | --- | --- |
| Preferred Attentional Focus | Control | External Focus | Internal Focus | Total |
| Control | 8 | 1 | 0 | 9 |
| External Focus | 2 | 6 | 0 | 8 |
| Internal Focus | 4 | 3 | 6 | 13 |
| Total | 14 | 10 | 6 | 30 |

***Day 2***

The preferred attentional focus and the perceived best attentional focus matched in 24 of the 30 participants (Table 4).

**Table 4**

*Preferred vs. perceived best attentional focus on Day 2. Values represent the number of participants*

|  | Perceived Best Attentional Focus | | |  |
| --- | --- | --- | --- | --- |
| Preferred Attentional Focus | Control | External Focus | Internal Focus | Total |
| Control | 6 | 2 | 0 | 8 |
| External Focus | 3 | 6 | 1 | 10 |
| Internal Focus | 0 | 0 | 12 | 12 |
| Total | 9 | 8 | 13 | 30 |

***Day 3***

The preferred attentional focus and the perceived best attentional focus matched in 22 of the 30 participants (Table 5).

**Table 5**

*Preferred vs. perceived best attentional focus on Day 3. Values represent the number of participants*

|  | Perceived Best Attentional Focus | | |  |
| --- | --- | --- | --- | --- |
| Preferred Attentional Focus | Control | External Focus | Internal Focus | Total |
| Control | 7 | 2 | 1 | 10 |
| External Focus | 3 | 9 | 2 | 14 |
| Internal Focus | 0 | 0 | 6 | 6 |
| Total | 10 | 11 | 9 | 30 |

***Day 4***

The preferred attentional focus and the perceived best attentional focus matched in 26 of the 30 participants (Table 6).

**Table 6**

*Preferred vs. perceived best attentional focus on Day 4. Values represent the number of participants*

|  | Perceived Best Attentional Focus | | |  |
| --- | --- | --- | --- | --- |
| Preferred Attentional Focus | Control | External Focus | Internal Focus | Total |
| Control | 9 | 0 | 2 | 11 |
| External Focus | 1 | 8 | 0 | 9 |
| Internal Focus | 1 | 0 | 9 | 10 |
| Total | 11 | 8 | 11 | 30 |
